# Altered plasma protein profiles in genetic FTD – a GENFI study

**DOI:** 10.1186/s13024-023-00677-6

**Published:** 2023-11-15

**Authors:** Abbe Ullgren, Linn Öijerstedt, Jennie Olofsson, Sofia Bergström, Julia Remnestål, John C. van Swieten, Lize C. Jiskoot, Harro Seelaar, Barbara Borroni, Raquel Sanchez-Valle, Fermin Moreno, Robert Laforce, Matthis Synofzik, Daniela Galimberti, James B. Rowe, Mario Masellis, Maria Carmela Tartaglia, Elizabeth Finger, Rik Vandenberghe, Alexandre de Mendonça, Pietro Tirabosch, Isabel Santana, Simon Ducharme, Chris R. Butler, Alexander Gerhard, Markus Otto, Arabella Bouzigues, Lucy Russell, Imogen J. Swift, Aitana Sogorb-Esteve, Carolin Heller, Jonathan D. Rohrer, Anna Månberg, Peter Nilsson, Caroline Graff, on behalf of the Genetic Frontotemporal Dementia Initiative (GENFI), Sónia Afonso, Maria Rosario Almeida, Sarah Anderl-Straub, Christin Andersson, Anna Antonell, Andrea Arighi, Mircea Balasa, Myriam Barandiaran, Nuria Bargalló, Robart Bartha, Benjamin Bender, Emanuele Buratti, Luisa Benussi, Maxime Bertoux, Giuliano Binetti, Sandra Black, Martina Bocchetta, Sergi Borrego-Ecija, Jose Bras, Rose Bruffaerts, Marta Cañada, Valentina Cantoni, Paola Caroppo, David Cash, Miguel Castelo-Branco, Rhian Convery, Thomas Cope, Vincent Deramecourt, Giuseppe Di Fede, Alina Díez, Chiara Fenoglio, Catarina B. Ferreira, Nick Fox, Morris Freedman, Giorgio Fumagalli, Aurélie Funkiewiez, Alazne Gabilondo, Roberto Gasparotti, Serge Gauthier, Antonella Alberici, Giorgio Giaccone, Ana Gorostidi, Caroline Greaves, Rita Guerreiro, Begoña Indakoetxea, Vesna Jelic, Hans-Otto Karnath, Ron Keren, Gregory Kuchcinski, Tobias Langheinrich, Thibaud Lebouvier, Maria João Leitão, Albert Lladó, Carolina Maruta, Lieke Meeter, Gabriel Miltenberger, Rick van Minkelen, Sara Mitchell, Katrina Moore, Jennifer Nicholas, Jaume Olives, Sebastien Ourselin, Alessandro Padovani, Jessica Panman, Janne M. Papma, Georgia Peakman, Michela Pievani, Yolande Pijnenburg, Enrico Premi, Sara Prioni, Rosa Rademakers, Veronica Redaelli, Daisy Rinaldi, Tim Rittman, Ekaterina Rogaeva, Adeline Rollin, Pedro Rosa-Neto, Giacomina Rossi, Martin Rosser, Elio Scarpini, Elisa Semler, Rachelle Shafei, Christen Shoesmith, Miguel Tábuas-Pereira, Mikel Tainta, Ricardo Taipa, David Tang-Wai, David L. Thomas, Paul Thompson, Håkan Thonberg, Carolyn Timberlake, Emily Todd, Philip Van Damme, Mathieu Vandenbulcke, Michele Veldsman, Ana Verdelho, Jorge Villanua, Jason Warren, Carlo Wilke, Ione Woollacott, Henrik Zetterberg, Miren Zulaica, João Durães, Marisa Lima, João Lemos

**Affiliations:** 1 Swedish FTD Initiative, Stockholm, Sweden; 2 Department of Neurobiology, Care Sciences and Society, Division of Neurogeriatrics, Karolinska Institutet, Solna, Sweden; 3 Unit for Hereditary Dementias, Karolinska University Hospital, Solna, Sweden; 4 Department of Protein Science, Division of Affinity Proteomics, SciLifeLab, KTH Royal Institute of Technology, Stockholm, Sweden; 5 Department of Neurology, Erasmus Medical Centre, Rotterdam, Netherlands; 6 Department of Clinical and Experimental Sciences, Centre for Neurodegenerative Disorders, University of Brescia, Brescia, Italy; 7 Alzheimer’s Disease and Other Cognitive Disorders Unit, Neurology Service, Hospital Clínic, Institut d’Investigacións Biomèdiques August Pi I Sunyer, University of Barcelona, Barcelona, Spain; 8 Department of Neurology, Cognitive Disorders Unit, Donostia University Hospital, San Sebastian, Gipuzkoa, Spain; 9 Neuroscience Area, Biodonostia Health Research Institute, San Sebastian, Gipuzkoa, Spain; 10 Département Des Sciences Neurologiques, Clinique Interdisciplinaire de Mémoire, CHU de Québec, and Faculté de Médecine, Université Laval, Quebec City, QC Canada; 11 Department of Neurodegenerative Diseases, Hertie-Institute for Clinical Brain Research and Center of Neurology, University of Tübingen, Tübingen, Germany; 12 Center for Neurodegenerative Diseases (DZNE), Tübingen, Germany; 13 Fondazione IRCCS Ospedale Policlinico, Milan, Italy; 14 University of Milan, Centro Dino Ferrari, Milan, Italy; 15 Department of Clinical Neurosciences, University of Cambridge, Cambridge, UK; 16 Sunnybrook Health Sciences Centre, Sunnybrook Research Institute, University of Toronto, Toronto, Canada; 17 Tanz Centre for Research in Neurodegenerative Diseases, University of Toronto, Toronto, Canada; 18 Department of Clinical Neurological Sciences, University of Western Ontario, London, ON Canada; 19 Department of Neurosciences, Laboratory for Cognitive Neurology, KU Leuven, Leuven, Belgium; 20 Neurology Service, University Hospitals Leuven, Leuven, Belgium; 21 Leuven Brain Institute, KU Leuven, Leuven, Belgium; 22 Faculty of Medicine, University of Lisbon, Lisbon, Portugal; 23 Fondazione IRCCS Istituto Neurologico Carlo Besta, Milano, Italy; 24 Faculty of Medicine, University Hospital of Coimbra (HUC), Neurology Service, University of Coimbra, Coimbra, Portugal; 25 Center for Neuroscience and Cell Biology, Faculty of Medicine, University of Coimbra, Coimbra, Portugal; 26 Department of Psychiatry, McGill University Health Centre, McGill University, Montreal, Québec Canada; 27 McConnell Brain Imaging Centre, Montreal Neurological Institute, McGill University, Montreal, Québec Canada; 28 Nuffield Department of Clinical Neurosciences, Medical Sciences Division, University of Oxford, Oxford, UK; 29 Department of Brain Sciences, Imperial College London, London, UK; 30 Division of Neuroscience and Experimental Psychology, Wolfson Molecular Imaging Centre, University of Manchester, Manchester, UK; 31 Departments of Geriatric Medicine and Nuclear Medicine, Center for Translational Neuro- and Behavioral Sciences, University Medicine Essen, Essen, Germany; 32 Department of Neurology, University of Ulm, Ulm, Germany; 33 Department of Neurodegenerative Disease, Dementia Research Centre, UCL Institute of Neurology, Queen Square, London, UK; 34 Dementia Research Institute at UCL, UCL Queen Square Institute of Neurology, London, UK

**Keywords:** Frontotemporal dementia, Plasma biomarkers, *GRN*, *C9orf72*, *MAPT*, Neurodegeneration

## Abstract

**Background:**

Plasma biomarkers reflecting the pathology of frontotemporal dementia would add significant value to clinical practice, to the design and implementation of treatment trials as well as our understanding of disease mechanisms. The aim of this study was to explore the levels of multiple plasma proteins in individuals from families with genetic frontotemporal dementia.

**Methods:**

Blood samples from 693 participants in the GENetic Frontotemporal Dementia Initiative study were analysed using a multiplexed antibody array targeting 158 proteins.

**Results:**

We found 13 elevated proteins in symptomatic mutation carriers, when comparing plasma levels from people diagnosed with genetic FTD to healthy non-mutation controls and 10 proteins that were elevated compared to presymptomatic mutation carriers.

**Conclusion:**

We identified plasma proteins with altered levels in symptomatic mutation carriers compared to non-carrier controls as well as to presymptomatic mutation carriers. Further investigations are needed to elucidate their potential as fluid biomarkers of the disease process.

**Supplementary Information:**

The online version contains supplementary material available at 10.1186/s13024-023-00677-6.

## Background

Frontotemporal dementia (FTD) is a group of neurodegenerative diseases where the most common phenotypes are behavioural variant FTD (bvFTD) and primary progressive aphasias (PPA). There is a great heterogeneity in FTD, both in terms of clinical symptoms, underlying genetic causes, and neuropathological findings. Over the past years, effort has been put into explaining the diversity by searching for fluid biomarkers that reflect different aspects of FTD [1]. Most efforts have focused on finding biomarkers in cerebrospinal fluid (CSF) and a few promising candidates have been found, such as neurofilament light chain (NEFL) and neuronal pentraxin 2 (NPTX2) [2, 3]. However, the use of CSF biomarkers is limited by the invasive nature of the sampling procedure and restricted availability. Therefore, a reliable blood-based biomarker would be extremely valuable. A well-known blood-based biomarker in genetic FTD is progranulin (GRN), which is reduced in individuals with loss-of-function mutations in the gene with the same name [4]. While serum or plasma GRN levels can be used to confirm mutations in *GRN*, they do not correlate with clinically important metrics such as age at onset [4]. Previous studies have also identified glial fibrillary acidic protein (GFAP), tau and NEFL as possible plasma-based biomarkers, where GFAP is elevated in symptomatic *GRN* mutation carriers, tau is elevated in sporadic FTD and in symptomatic *MAPT* mutation carriers, and NEFL is elevated in both genetic and sporadic FTD [5–7]. However, none of the proteins are specific for FTD since increased levels have been observed in other neurological diseases [8, 9]. A large screen of plasma proteins in FTD and Alzheimer disease (AD) found a panel of 12 proteins that discriminated between the two diseases. However, these proteins were associated with AD pathology and no differences were found between FTD cases and controls [10]. Further studies are therefore needed to find biomarkers that are FTD specific.

Here, we present an exploratory plasma profiling study of 158 proteins in 693 participants in the well-described genetic FTD cohort. To our knowledge, a plasma proteomic analysis of this magnitude has not been done in genetic FTD before. We aimed to investigate differences in plasma protein levels between both symptomatic and presymptomatic mutation carriers compared to non-carrier family members who serve as controls. Our findings indicate alterations in plasma protein levels between symptomatic mutation carriers and non-carrier controls, as well as gene specific differences in *GRN* mutation carriers.

## Materials and methods

### Cohort

All clinical data and samples included in the study were collected within the Genetic frontotemporal dementia initiative (GENFI) between 2012 and 2019 [11]. Variables included were age at sampling, sex, mutation group (symptomatic mutation carriers, SMC; presymptomatic mutation carriers, PMC; or non-carrier controls, NC), genetic group (chromosome 9 open reading frame 72, *C9orf72;* progranulin, *GRN;* or microtubule associated protein tau, *MAPT*), clinical phenotype, and age at onset. In total, baseline plasma samples from 701 participants were collected including 141 SMC (63 *C9orf72*, 50 *GRN*, and 28 *MAPT*), 283 PMC (97 *C9orf72*, 135 *GRN* and 51 *MAPT*) and 277 NC. Carriers of FTD-causing variants in other genes were not included. Clinically, the SMC most frequently presented with bvFTD (*n* = 102), followed by PPA (*n* = 25), FTD with concomitant amyotrophic lateral sclerosis (ALS) (*n* = 5) and other FTD-related phenotypes (*n* = 5).

### Sample collection according to GENFI protocol

Blood samples (*n* = 701) were collected at 20 different sites in Europe and Canada in ethylenediaminetetraacetic acid (EDTA) tubes. Samples were centrifuged at 2200 × g for 5 min at 22 °C and the supernatant plasma was transferred to 0.5 ml polypropylene cryotubes and stored at -80 °C until analysis.

### Suspension bead array assay

The plasma samples were diluted and labelled with a tenfold molar excess of biotin (NHS-PEG4-biotin. 21329, Thermo Scientific), heat treated, and subsequently mixed with an antibody suspension bead array as described in detail previously [12, 13]. A streptavidin conjugated fluorophore (Streptavidin R-Phycoerythrin Conjugate, Invitrogen) enabled the detection of the proteins, and the readout was performed on a Flexmap 3D instrument (Luminex corporation). Binding events were displayed as signal intensity. Published as well as internal unpublished work were used to guide the selection of target proteins (*n* = 163) which was based on previously identified promising targets, proteins involved in suggested pathological processes of neurodegeneration and proteins with enriched expression in brain compared to other tissue [14–17]. The majority of the antibodies (*n* = 156) were selected from the Human Protein Atlas project (www.proteinatlas.org) and the remaining seven were obtained from other providers (M067-3 from MBL International; MA1-70053, PA5-34943, 34–1000 from Invitrogen Antibodies; MAB2037-SP, AF2420, AF3154 from R&D Systems). The mean coefficient of variance per 384-well plate (*n* = 3) was less than 10%, and 97% of the antibodies had an individual coefficient of variance below 20%. The inter-assay correlations were high (rho > 0.8 for 154 antibodies). After quality control analysis, five antibodies were excluded due to high correlation to a negative control (rho > 0.6) resulting in 158 protein targets for further analysis (Supplementary Table 1).

### Statistical analysis

#### Data pre-processing

All data pre-processing, analysis and illustrations were performed in R Studio version 2022.2.3.492 using R version 4.2.1 [18]. The data was normalised in two steps to diminish the effects of time delay during readout and potential differences between plates [19]. Prior to statistical analysis, the data was log2- and z-transformed via mean centring and unit variance scaling. Outlier samples with a median protein level three standard deviations higher or lower than the median for the whole cohort were excluded from the analysis (*n* = 8 samples removed). A residual adjustment approach was used to deal with the potential confounding effect of healthy ageing on protein levels [20]. The effect of healthy ageing on protein levels was estimated in the NC via linear mixed effect models using protein levels as the response variable, age as a fixed effect and collection site as a random intercept (lmer, lme4, [21]). For each subject in the overall cohort (including each of the NC, PMC, and SMC groups), the adjusted protein levels were then obtained through the following:

$${Protein}_{adj.}=Protein- \beta ( Age-\overline{Age} )$$

where Protein_adj._ is the age adjusted protein level, Protein is the original protein level, β is the age-associated beta coefficient, Age is the subject’s age and $\overline{Age}$ is the mean age in the entire cohort.

#### Demographic statistics

The participants’ ages followed a normal distribution, evaluated by visual assessment of normal probability plot and histogram. Differences in age between SMC, PMC and NC were assessed by one-way ANOVA and Tukey’s HSD post hoc test. Pearson’s Chi-squared test was used to investigate differences in sex between SMC, PMC and NC. *P*-values below 0.05 were considered significant.

#### Protein profile analysis

Differences in protein levels between SMC and NC were examined via binomial generalised linear mixed effects models using clinical status (i.e. SMC or NC) as the response variable, protein levels and sex as fixed effects with a random intercept based on collection site (glmer, lme4, [21]). One model per protein was built. The same method was used to assess differences in protein levels between SMC and presymptomatic mutation carriers (PMC), PMC versus NC, as well as to analyse gene specific differences e.g., SMC carrying a *GRN* mutation (SMC-GRN) vs NC. Log2 fold changes were calculated by subtracting the median log2 transformed protein levels in NC or PMC from the median log2 transformed protein levels in SMC. In contrast, the effects of age and sex on the protein levels in mutation carriers as well as in non-carriers were estimated via generalised linear mixed-effects models using protein levels as the response; age and sex as fixed effects with a random intercept based on collection site (lmer, lme4, [21]). *P*-values were calculated using the Satterthwaite’s degrees of freedom method (lmerTest, [22]). Multiple testing corrections were made via the Benjamini–Hochberg method for controlling false-discovery rates and an adjusted *p*-value of 0.05 was considered significant. Only adjusted *p*-values are reported unless clearly stated otherwise. Protein – protein correlations were calculated using Spearman's rank correlation coefficient. Protein clusters are based on hierarchical clustering using Ward’s clustering criterion.

## Results

### Cohort

In total, plasma results from 693 participants were included in the statistical analysis, and demographic data of the cohort is presented in Table 1. The age was not significantly different between NC and PMC (*p*-value = 0.06), but SMC were older than both NC and PMC (*p*-value = 7.13 × 10^–41^). There were more females in NC (55%) and PMC (62%) compared to SMC (42%) (NC vs SMC: *p*-value = 1.3 × 10^–2^; PMC vs SMC: *p*-value = 1.1 × 10^–4^).

**Table 1:** Demographic data of the cohort

	**Non-carriers (NC)**	**Presymptomatic mutation carriers (PMC)**	**Symptomatic mutation carriers (SMC)**	**Total**	***p*-value**
No. of participants	276	280	137	693	
Age, mean years (SD)	47 (14)	45 (12)	63 (9)	49 (14)	< 0.001^a^
Females (%)	152 (55)	174 (62)	57 (42)	383 (55)	< 0.001^b^
Mutated gene (%)				417 (60)	
* GRN*		133 (48)	49 (36)		
* C9orf72*		96 (34)	62 (45)		
* MAPT*		51 (18)	26 (19)		
Age at onset, mean years (SD)					
* GRN*			61 (8)		
* C9orf72*			60 (9)		
* MAPT*			51 (8)		

^a^One-way ANOVA (F(2,690) = 106.0, *p* = 7.13 × 10^–41^). Differences in age between NC vs SMC and PMC vs SMC (Tukey multiple comparison post-hoc test). No difference between NC and PMC (*p* = 0.06)

^b^Pearson’s Chi-squared test. More females in NC and PMC compared to SMC (X^2^(1, *N* = 413) = 6.1, *p* = 1.3 × 10^–2^) and X^2^(1, *N* = 417) = 14.9, *p* = 1.1 × 10^–4^ respectively). No difference between NC and PMC (X^2^(1, *N* = 556) = 2.6, *p* = 0.1)

### Altered plasma protein levels in symptomatic mutation carriers

**Fig. 1:**
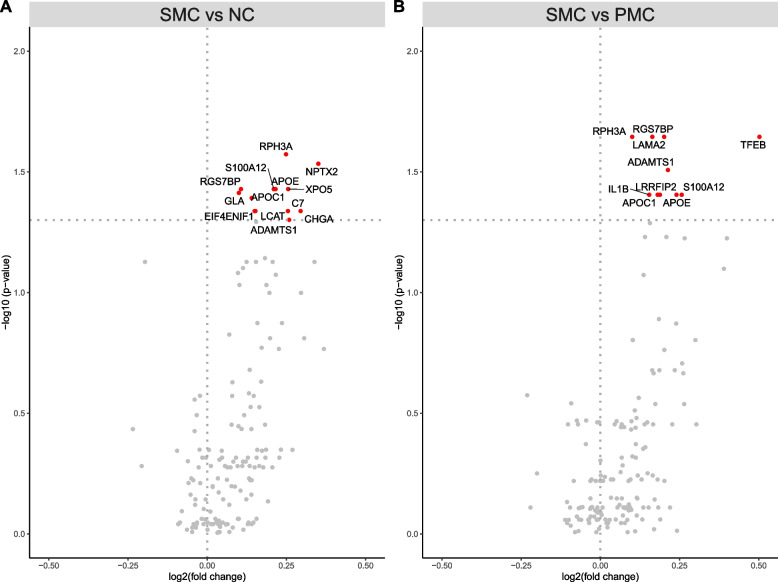
Volcano plots of plasma protein levels showing -log10 (*p*-values) of the log2(fold change) for comparisons between different groups. Plasma protein level differences between A) all SMC and NC, B) all SMC and all PMC. Each protein is represented by a gray dot and are coloured red if the protein levels are increased in the SMC compared to the comparison group (NC, or PMC). Dotted horizontal line = adjusted *p*-value 0.05, dotted vertical line = log2 fold change 0

**Fig. 2:**
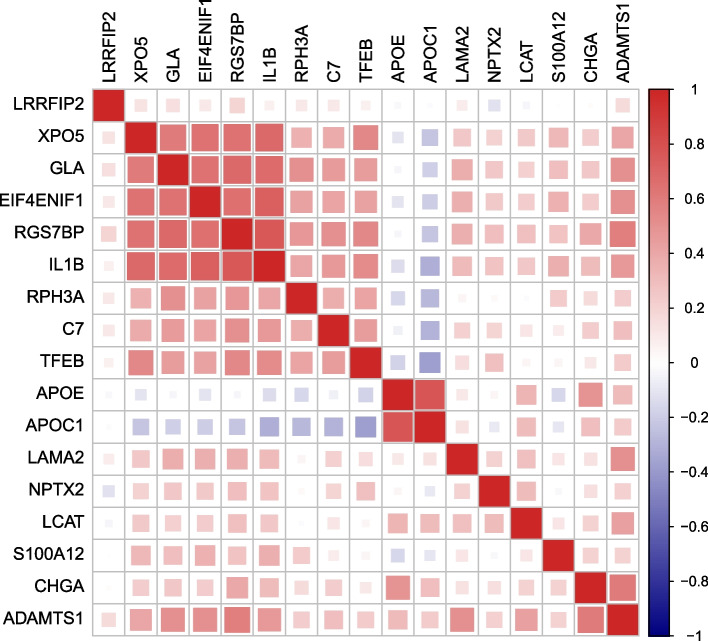
Plots of protein – protein correlations. Protein order is based on hierarchical clustering. Correlation plot of the 17 proteins with elevated levels in symptomatic mutation carriers (SMC) compared to non-carriers (NC) or presymptomatic mutation carriers (PMC). The colour scale indicates Spearman's rank correlation coefficient from dark blue (-1) to bright red (1)

When comparing plasma protein levels in SMC to NC, we found that 13 proteins were elevated in SMC (Fig. 1A, Table 2 and Supplementary Fig. 1). In the comparison between SMC versus PMC, we found that 10 proteins were elevated in SMC (Fig. 1B, Table 2). There were six overlapping proteins i.e., six proteins had elevated levels in SMC both in the comparison to NC as well as in the comparison to the protein levels in PMC. An overview of how these proteins correlate with each other can be found in Fig. 2. When stratifying by genetic group, rabphilin 3a (RPH3A) was increased in SMC-GRN compared to NC (*p*-value = 1.3 × 10^–3^, odds ratio = 1.915) whereas progranulin, as expected, was decreased (*p*-value = 9.3 × 10^–6^, odds ratio = 0.152). No proteins had significantly different levels in the comparison between SMC-C9 and NC nor in the comparison between SMC-MAPT and NC (data not shown).

**Table 2:** Comparison of plasma protein levels in symptomatic mutation carriers versus non-carriers and presymptomatic mutation carriers

*Protein*	*SMC vs NC*		*SMC vs PMC*	
	*p*-value	Odds ratio	*p*-value	Odds ratio
RPH3A*	2.68 × 10^–2^	1.535 (1.228—1.919)	2.27 × 10–^2^	1.46 (1.177—1.811)
NPTX2	2.93 × 10^–2^	1.499 (1.2—1.874)	*3.52* × *10*^*–1*^	*1.176 (0.964—1.433)*
XPO5	3.73 × 10^–2^	1.598 (1.216—2.1)	*1.29* × *10*^*–1*^	*1.322 (1.056—1.656)*
RGS7BP*	3.73 × 10^–2^	1.504 (1.181 – 1.915)	2.27 × 10^–2^	1.619 (1.246 – 2.103)
APOE*	3.73 × 10^–2^	1.532 (1.179—1.992)	3.94 × 10^–2^	1.516 (1.165—1.972)
S100A12*	3.73 × 10^–2^	1.5 (1.173—1.919)	3.94 × 10^–2^	1.394 (1.126—1.725)
GLA	3.87 × 10^–2^	1.605 (1.194—2.158)	*4.25* × *10*^*–1*^	*1.185 (0.938—1.498)*
APOC1*	4.06 × 10^–2^	1.627 (1.194—2.218)	3.94 × 10^–2^	1.651 (1.2—2.271)
EIF4ENIF1	4.60 × 10^–2^	1.503 (1.144—1.976)	*3.31* × *10*^*–1*^	*1.264 (0.976—1.636)*
LCAT	4.60 × 10^–2^	1.414 (1.128—1.773)	*8.46* × *10*^*–2*^	*1.336 (1.075—1.662)*
C7	4.60 × 10^–2^	1.49 (1.14—1.948)	*3.08* × *10*^*–1*^	*1.277 (0.981—1.663)*
CHGA	4.60 × 10^–2^	1.458 (1.132—1.877)	*5.96* × *10*^*–2*^	*1.436 (1.111—1.855)*
ADAMTS1*	5.00 × 10^–2^	1.404 (1.113—1.77)	3.11 × 10^–2^	1.564 (1.198—2.04)
TFEB	*7.47* × *10*^*–2*^	*1.362 (1.082—1.715)*	2.27 × 10^–2^	1.555 (1.229—1.967)
LRRFIP2	*7.47* × *10*^*–2*^	*1.344 (1.079—1.674)*	3.94 × 10^–2^	1.44 (1.141—1.818)
LAMA2	*9.31* × *10*^*–2*^	*1.327 (1.057—1.667)*	2.27 × 10^–2^	1.57 (1.219—2.022)
IL1B	*1.69* × *10*^*–1*^	*1.308 (1.018—1.681)*	3.94 × 10^–2^	1.576 (1.173—2.117)

Proteins with statistically significant different plasma levels in the comparison between symptomatic mutation carriers (SMC) and non-carriers (NC) or in the comparison between SMC and presymptomatic mutation carriers (PMC), including *p*-values and odds ratios with 95% confidence intervals. All *p*-values are adjusted for multiple testing. Non-significant *p*-values are in italics. An asterisk indicates proteins with significantly different plasma levels in both comparisons

Next, we investigated the correlation between age and protein levels of the altered proteins in mutation carriers. Among the 17 unique proteins with elevated levels in SMC (SMC vs NC or SMC vs PMC), 13 had a significant correlation with age (Table 3).

**Table 3:** Correlations between age and protein levels in mutation carriers

*Protein*	*p-value*	*β*
RPH3A	9.98 × 10^–7^	0.019 (0.013—0.026)
IL1B	2.16 × 10^–4^	0.012 (0.006—0.017)
RGS7BP	5.35 × 10^–4^	0.012 (0.006—0.018)
TFEB	5.35 × 10^–4^	0.013 (0.006—0.019)
S100A12	1.04 × 10^–3^	0.013 (0.006—0.02)
GLA	4.18 × 10^–3^	0.01 (0.004—0.016)
EIF4ENIF1	5.22 × 10^–3^	0.008 (0.003—0.014)
APOE	5.22 × 10^–3^	0.009 (0.003—0.015)
CHGA	5.22 × 10^–3^	0.009 (0.003—0.015)
LRRFIP2	5.22 × 10^–3^	0.011 (0.004—0.018)
ADAMTS1	5.25 × 10^–3^	0.009 (0.003—0.015)
LAMA2	2.15 × 10^–2^	0.008 (0.002—0.014)
APOC1	3.09 × 10^–2^	0.006 (0.001—0.011)
XPO5	*1.19* × *10*^*–1*^	*0.006 (-0.001—0.013)*
LCAT	*1.57* × *10*^*–1*^	*0.005 (-0.002—0.012)*
NPTX2	*1.68* × *10*^*–1*^	*0.005 (-0.002—0.013)*
C7	*3.73* × *10*^*–1*^	*0.003 (-0.003—0.008)*

Correlations between protein levels and age in all mutation carriers (MC) including *p*-values and beta coefficients with 95% confidence intervals. All *p*-values are adjusted for multiple testing. Non-significant *p*-values are in italics

When analysing sex differences for the 17 proteins elevated in SMC, two proteins were found to be increased in men compared to women in the mutation carriers: apolipoprotein E (APOE, *p*-value = 1.73 × 10^–2^, β = 0.267) and apolipoprotein C1 (APOC1, *p*-value = 1.73 × 10^–2^, β = 0.235). No sex differences were found among the NC for either protein (*p*-value = 1.49 × 10^–1^ and *p*-value = 7.4 × 10^–1^, respectively).

Finally, we explored if any of the 158 proteins included in this study were found at different levels in SMC with bvFTD compared to SMC with PPA but found no significant differences (data not shown).

### Plasma protein levels in the presymptomatic stage

We also investigated the possibility to detect differences in protein levels already at the presymptomatic stage of FTD by first comparing PMC to NC and then stratifying by gene. The only difference found in these comparisons was decreased levels of GRN in PMC-GRN compared to NC (*p*-value = 4.44 × 10^–3^). However, the proteins neurofilament medium chain (NEFM), neuronal pentraxin 2 (NPTX2) and chitinase 3 like 1 (CHI3L1) showed trends of being elevated in PMC-GRN compared to NC (unadjusted *p*-value = 3.1 × 10^–3^, unadjusted *p*-value = 4.8 × 10^–3^ and unadjusted *p*-value = 4.6 × 10^–3^, respectively), though these differences were not significant after adjustment for multiple testing. None of the three proteins showed any correlation with age when analysed in PMC-GRN alone or in PMC-GRN together with SMC-GRN (Supplementary Table 2). NPTX2 was, however, elevated in SMC compared to NC and CHI3L1 was just above the significance threshold in the same comparison (*p*-value = 5.1 × 10^–2^). Neither were significant in the comparison between SMC and PMC (both *p*-values > 0.3).

## Discussion

We performed extensive protein profiling of plasma from a genetic FTD cohort, collected within the GENFI study. We found 13 significantly increased plasma proteins in patients with genetic FTD compared to non-carrier controls and 10 proteins that were significantly increased compared to presymptomatic mutation carriers. Six of these proteins were significantly different in both comparisons, indicating that they likely are associated with symptom onset rather than the presence of one of the pathogenic mutations. These six proteins were also significantly correlated with increased age in mutation carriers, after correcting for healthy ageing, which further strengthens their association with symptom onset.

In contrast, four proteins, increased in SMC vs NC, were not correlated with age, nor elevated in the SMC vs PMC comparison, suggesting that they may be elevated already before symptom onset. One of these proteins NPTX2, is of particular interest. NPTX2, a synaptic protein, which has previously been shown to be reduced in CSF from patients with FTD and is potentially one of the first protein biomarkers to become abnormal in genetic FTD [3, 23, 24]. We have, as of yet, no explanation to why NPTX2 is reduced in CSF and elevated in plasma, or if the NPTX2 detected in plasma is brain derived. However, finding elevated levels of NPTX2 in plasma from SMC suggests that NPTX2 could work as a plasma-based biomarker.

We found two proteins, GRN and RPH3A, that differed in SMC-GRN compared to NC, while no proteins were observed at different levels in neither SMC-MAPT nor SMC-C9, compared to NC. A reduction of GRN in progranulin mutation carriers is of course expected since all known pathogenic FTD-related *GRN* mutations lead to haploinsufficiency. On the other hand, RPH3A was elevated in SMC-GRN. RPH3A is involved in presynaptic vesicle trafficking and has been implicated to play a role in synaptic dysfunction in other neurodegenerative diseases [25, 26]. In addition to the findings in SMC-GRN, we observed some indications of differences already in the presymptomatic stages in *GRN* mutation carriers. While not statistically significant after adjustment for multiple testing, the differences are still noteworthy since the proteins, NEFM, NPTX2 and CHI3L1, all have been reported as biomarker candidates in CSF [16, 27]. NPTX2 was also elevated in all SMC compared to NC and CHI3L1 was just above the threshold for significance while neither of these two proteins were elevated in SMC when compared to PMC. Taken together this indicates that these two proteins might be upregulated already at the presymptomatic stage. However, further studies are needed to establish if these proteins indeed are related to presymptomatic changes in *GRN* mutation carriers or if it is a spurious finding.

Biological sex is a known risk factor for several types of dementia, with female sex being a risk factor for AD and male sex being more common in FTD [28, 29]. In light of this, we analysed if any of the proteins identified in the current study exhibited any sex specific patterns. While we could not determine any significant interactions between sex and mutation status for these proteins (data not shown), two proteins were significantly correlated with sex in the mutation carrier group, but not in controls, suggesting a potential biological effect.

We acknowledge several limitations in this study. The focus was on genetic FTD and samples from patients with other neurodegenerative diseases were not included in the analysis. Follow-up studies with comparisons to for example AD and ALS will elucidate the importance of altered plasma proteins in FTD in relation to other diseases as well as sporadic FTD. The suspension bead array technique is a method for analysing multiple proteins simultaneously, which is useful in an exploratory study like this. However, a high-throughput antibody-based single-binder assay can have reduced sensitivity, which may limit the detection of low abundant proteins and require further validation of antibody specificity. In addition, we used a targeted approach, and the protein analysis was thus limited by the protein selection as well as the availability of antibodies.

## Conclusions

To our knowledge, this is the first large scale plasma protein profiling specifically in genetic FTD. A reliable fluid biomarker could aid for example in diagnosing FTD at an early stage or in selecting individuals for upcoming clinical trials. Blood-based biomarkers would have the advantage of being easy to access and widely available compared to CSF-biomarkers. Here, we have presented an exploratory study providing proteins, including a previous CSF-biomarker, that are of interest for future investigations as potential biomarkers.

### Supplementary Information

**Additional file 1: Supplementary Table 1. **Antibodies used in the suspension bead array plasma analysis. **Supplementary Table 2.** Proteins with different levels in PMC compared to NC. **Supplementary Figure 1. **Boxplots for the 13 proteins that differed between SMC and NC. **Supplementary Figure 2.** Boxplots for the 10 proteins that differed between SMC and PMC.

## Data Availability

Anonymized data may be shared upon request from a qualified academic investigator for the purpose of replication of the results and procedures detailed in this article. All requests must be in agreement with EU legislation on general data protection and must be in line with the decisions from the Ethical Review Board of Sweden. Data sharing should be regulated in a material transfer agreement and/or data processing agreement as appropriate.
