# Supplementary material for: Altered plasma protein profiles in genetic FTD – a GENFI study
Source: Mol Neurodegener. 2023 Nov 15;18:85. doi: 10.1186/s13024-023-00677-6 (PMC10648335; doi:10.1186/s13024-023-00677-6)

# SUPPLEMENTARY MATERIALS

Altered plasma protein profiles in genetic FTD – a GENFI study

Abbe Ullgren^1,2,#^, Linn Öijerstedt^1,2,3,#^, Jennie Olofsson^1,4^, Sofia Bergström^1,4^, Julia Remnestål^1,4^, John C. van Swieten^5^, Lize C, Jiskoot^5^, Harro Seelaar^5^, Barbara Borroni^6^, Raquel Sanchez-Valle^7^, Fermin Moreno^8,9^, Robert Laforce^10^, Matthis Synofzik^11,12^, Daniela Galimberti^13,14^, James B. Rowe^15^, Mario Masellis^16^, Maria Carmela Tartaglia^17^, Elizabeth Finger^18^, Rik Vandenberghe^19,20,21^, Alexandre de Mendonça^22^, Pietro Tirabosch^23^, Isabel Santana^24,25^, Simon Ducharme^26,27^, Chris R. Butler^28,29^, Alexander Gerhard^30,31^, Markus Otto^32^, Arabella Bouzigues^33,34^, Lucy Russell^33,34^, Imogen J. Swift^33,34^, Aitana Sogorb-Esteve^33,34^, Carolin Heller^33,34^, Jonathan D. Rohrer^33,34^, Anna Månberg^1,4^ , Peter Nilsson^1,4^, Caroline Graff^1,2,3^, on behalf of the Genetic Frontotemporal Dementia Initiative (GENFI)

AFFILIATIONS

1. Swedish FTD Initiative, Stockholm, Sweden
2. Department of Neurobiology, Care Sciences and Society, Division of Neurogeriatrics, Karolinska Institutet, Solna, Sweden
3. Unit for Hereditary Dementias, Karolinska University Hospital, Solna, Sweden
4. Division of Affinity Proteomics, Department of Protein Science, SciLifeLab, KTH Royal Institute of Technology, Stockholm, Sweden
5. Department of Neurology, Erasmus Medical Centre, Rotterdam, Netherlands
6. Centre for Neurodegenerative Disorders, Department of Clinical and Experimental Sciences, University of Brescia, Italy
7. Alzheimer’s disease and Other Cognitive Disorders Unit, Neurology Service, Hospital Clínic, Institut d’Investigacións Biomèdiques August Pi I Sunyer, University of Barcelona, Barcelona, Spain
8. Cognitive Disorders Unit, Department of Neurology, Donostia University Hospital, San Sebastian, Gipuzkoa, Spain
9. Neuroscience Area, Biodonostia Health Research Institute, San Sebastian, Gipuzkoa, Spain
10. Clinique Interdisciplinaire de Mémoire, Département des Sciences Neurologiques, CHU de Québec, and Faculté de Médecine, Université Laval, QC, Canada
11. Department of Neurodegenerative Diseases, Hertie-Institute for Clinical Brain Research and Center of Neurology, University of Tübingen, Tübingen, Germany
12. Center for Neurodegenerative Diseases (DZNE), Tübingen, Germany
13. Fondazione IRCCS Ospedale Policlinico, Milan, Italy
14. University of Milan, Centro Dino Ferrari, Milan, Italy
15. Department of Clinical Neurosciences, University of Cambridge, Cambridge, UK
16. Sunnybrook Health Sciences Centre, Sunnybrook Research Institute, University of Toronto, Toronto, Canada
17. Tanz Centre for Research in Neurodegenerative Diseases, University of Toronto, Toronto, Canada
18. Department of Clinical Neurological Sciences, University of Western Ontario, London, Ontario Canada
19. Laboratory for Cognitive Neurology, Department of Neurosciences, KU Leuven, Leuven, Belgium
20. Neurology Service, University Hospitals Leuven, Belgium
21. Leuven Brain Institute, KU Leuven, Leuven, Belgium
22. Faculty of Medicine, University of Lisbon, Lisbon, Portugal
23. Fondazione IRCCS Istituto Neurologico Carlo Besta, Milano, Italy
24. University Hospital of Coimbra (HUC), Neurology Service, Faculty of Medicine, University of Coimbra, Coimbra, Portugal
25. Center for Neuroscience and Cell Biology, Faculty of Medicine, University of Coimbra, Coimbra, Portugal
26. Department of Psychiatry, McGill University Health Centre, McGill University, Montreal, Québec, Canada
27. McConnell Brain Imaging Centre, Montreal Neurological Institute, McGill University, Montreal, Québec, Canada
28. Nuffield Department of Clinical Neurosciences, Medical Sciences Division, University of Oxford, Oxford, UK
29. Department of Brain Sciences, Imperial College London, UK
30. Division of Neuroscience and Experimental Psychology, Wolfson Molecular Imaging Centre, University of Manchester, Manchester, UK
31. Departments of Geriatric Medicine and Nuclear Medicine, Center for Translational Neuro- and Behavioral Sciences, University Medicine Essen, Essen, Germany
32. Department of Neurology, University of Ulm, Ulm, Germany
33. Department of Neurodegenerative Disease, Dementia Research Centre, UCL Institute of Neurology, Queen Square, London, UK
34. UK Dementia Research Institute at UCL, UCL Queen Square Institute of Neurology, London, UK.

# Shared first author

## Supplementary Table 1. All antibodies used in the suspension bead array plasma analysis.

| Protein | Antibody | Protein | Antibody |
| --- | --- | --- | --- |
| AAAS | HPA070457 | LRP1 | HPA004182 |
| ACAN | HPA038242 | LRP5 | HPA003896 |
| ADAMTS1 | HPA068556 | LRRC4B | HPA058986 |
| AFM | HPA052437 | LRRFIP2 | HPA035956 |
| AP2B1 | HPA004921 | MAPK8IP2 | HPA034779 |
| APOA4 | HPA001352 | MAPT | HPA069570 |
| APOC1 | HPA051518 | MRC1 | HPA004114 |
| APOE | HPA068768 | RAB39A | HPA042505 |
| APOE4 | M067-3 | MYOC | HPA024314 |
| APOL1 | HPA018885 | NCAN | HPA077060 |
| AQP4 | HPA014944 | NEFH | HPA061615 |
| AXL | HPA037422 | NEFM | 34-1000 |
| AZGP1 | HPA012582 | NFKB1 | HPA027305 |
| BAG3 | HPA018493 | NPTX1 | HPA077062 |
| BEND6 | HPA052973 | NPTX2 | HPA049799 |
| C1QA | HPA002350 | NPTXR | HPA001079 |
| C3B | MA1-70053 | NRGN | HPA038171 |
| C4A | HPA046356 | NUP107 | HPA024141 |
| C5 | HPA075945 | NUP153 | HPA027898 |
| C5a | MAB2037-SP | NUP155 | HPA037774 |
| C7 | HPA067450 | NUP160 | HPA065113 |
| C8G | HPA046269 | NUP58 | HPA039360 |
| C9 | HPA029577 | NUPL2 | HPA049036 |
| CANX | HPA009681 | OLFML1 | HPA058824 |
| CARNS1 | HPA038570 | OLFML3 | HPA056362 |
| CASP14 | HPA027062 | OMG | HPA008206 |
| CAT | HPA051282 | P2RX7 | HPA034967 |
| CCK | HPA045039 | PDYN | HPA049841 |
| CCL22 | HPA077819 | PFN1 | HPA072376 |
| CD14 | HPA002035 | PGM2L1 | HPA056995 |
| CD163 | HPA046404 | PRKCA | HPA006564 |
| CD200R1 | HPA029009 | PRRT3 | HPA035127 |
| CD44 | HPA006080 | PSAP | HPA004426 |
| CD47 | HPA044659 | PTPRN2 | HPA026656 |
| CDH15 | HPA010700 | RANGAP1 | HPA062034 |
| CHGA | HPA017369 | RARS | HPA003979 |
| CHI3L1 | HPA077365 | RELN | HPA077891 |
| CHIT1 | HPA010575 | REST | HPA005784 |
| CHMP2B | HPA035069 | RGPD1 | HPA049497 |
| CLEC2L | HPA045050 | RGS7BP | HPA040349 |
| CLSTN3 | HPA070830 | RIC8A | HPA044221 |
| CNN2 | HPA049095 | RIPK3 | HPA055087 |
| CSE1L | HPA038059 | RNASE1 | HPA001140 |
| CTSL | HPA070413 | RPH3A | HPA002475 |
| CX3CL1 | HPA056729 | S100A12 | HPA003620 |
| CX3CR1 | HPA077743 | SEC62 | HPA014059 |
| DNAJB1 | HPA063247 | SEC63 | HPA053295 |
| DNAJC10 | HPA031111 | SEPT6 | HPA003459 |
| DRP2 | HPA002949 | SERPINA3 | HPA000893 |
| EIF4ENIF1 | HPA001619 | SERPINF1 | HPA076527 |
| F5 | HPA050035 | SERPING1 | HPA048738 |
| FAT2 | HPA072249 | SF3B1 | HPA054596 |
| FDPS | HPA028200 | SLITRK1 | HPA074835 |
| FGA | HPA064755 | SORT1 | AF3154 |
| FUS | HPA008717 | SPR | HPA039505 |
| GALNS | HPA042433 | SQOR | HPA017079 |
| GAP43 | PA5-34943 | ST6GAL2 | HPA014645 |
| GBA | HPA006667 | SYT5 | HPA010688 |
| GDA | HPA019352 | TARDBP | HPA070770 |
| GLA | HPA000966 | TBK1 | HPA050589 |
| GLUD1 | HPA044839 | TENM2 | HPA068691 |
| GLUL | HPA007316 | TFEB | HPA067082 |
| GRIA4 | HPA071597 | TLR4 | HPA068496 |
| GRN | AF2420 | TMEM119 | HPA052650 |
| HCFC1 | HPA018312 | TMEM132B | HPA035662 |
| HEXA | HPA075319 | TMEM235 | HPA053939 |
| HMOX1 | HPA000635 | TNF | HPA055037 |
| HSPA6 | HPA028549 | TNFSF10 | HPA054938 |
| IL1B | HPA075019 | TNR | HPA029859 |
| IL1RAP | HPA035293 | TRAPPC10 | HPA039419 |
| IL3 | HPA008412 | TREM2 | HPA012571 |
| IL6ST | HPA076931 | TRIM67 | HPA034776 |
| IPO7 | HPA019002 | UBL3 | HPA043917 |
| JUN | HPA059474 | VGF | HPA058371 |
| KIF5A | HPA004469 | VSTM2B | HPA073612 |
| KLF4 | HPA002926 | XPO5 | HPA018402 |
| LAMA2 | HPA003441 | XPOT | HPA048067 |
| LCAT | HPA044767 | YWHAE | HPA007924 |
| LRG1 | HPA001888 | YWHAH | HPA066376 |

## Supplementary Table 2. Left-hand side: proteins with statistically significant differences in protein levels between PMC-GRN and NC before adjustment for multiple testing, including unadjusted p-values and odds ratios with 95% confidence intervals. Right-hand side: correlations between protein levels and age in PMC-GRN, including p-values and beta coefficients with 95% confidence intervals. The p-values on the right-hand side are adjusted for multiple testing. Non-significant p-values are in italics.

| Protein | Group differences | | Age correlations | |
| --- | --- | --- | --- | --- |
|  | p-value | Odds ratio | p-value | β |
| GRN | 2.83×10^-5^ | 0.465 (0.325 - 0.666) | *6.67×10^-1^* | *-0.006 (-0.014 - 0.002)* |
| NEFM | 3.09×10^-3^ | 1.444 (1.132 - 1.843) | *9.44×10^-1^* | *0 (-0.011 - 0.01)* |
| CHI3L1 | 4.57×10^-3^ | 1.434 (1.118 - 1.84) | *9.44×10^-1^* | *-0.001 (-0.014 - 0.011)* |
| NPTX2 | 4.80×10^-3^ | 1.385 (1.104 - 1.736) | *9.44×10^-1^* | *0.003 (-0.008 - 0.014)* |

**
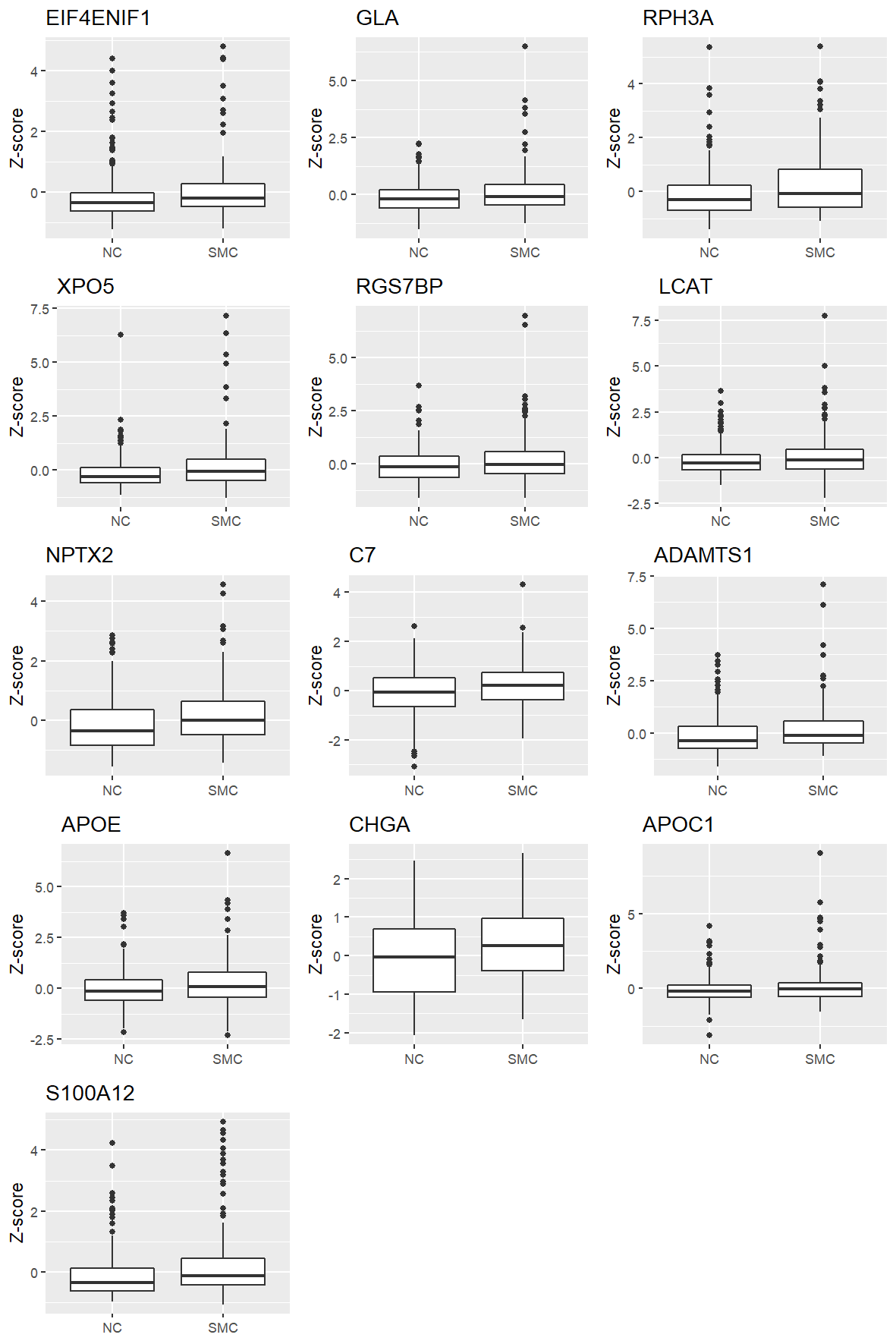
Supplementary Figure 1.** Boxplots for the 13 proteins that differed between SMC and NC.

**Supplementary figure 2.** Boxplots for the 10 proteins that differed between SMC and PMC.


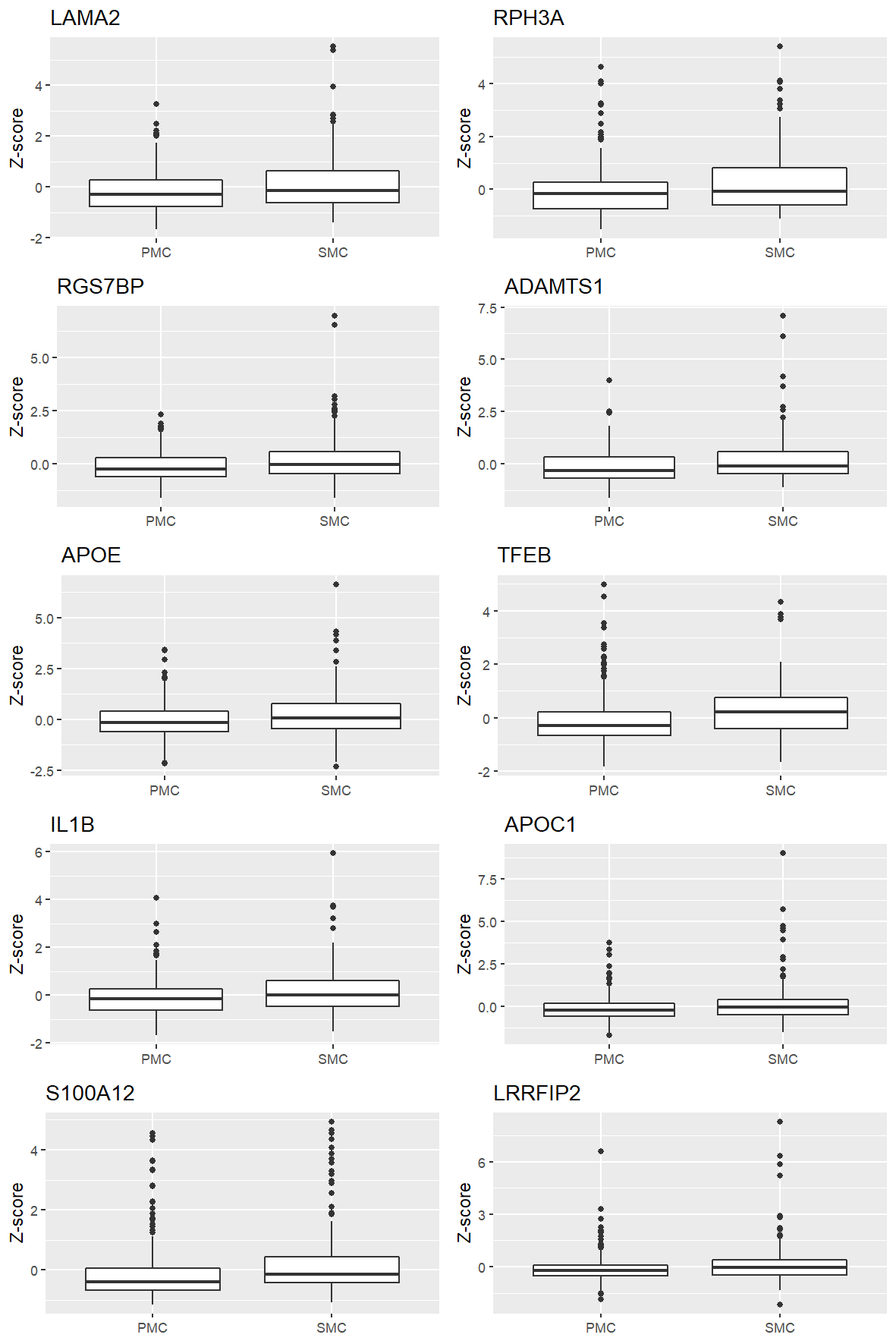

Supplement: Supplementary file 1 — Additional file 1: Supplementary Table 1. Antibodies used in the suspension bead array plasma analysis. Supplementary Table 2. Proteins with different levels in PMC compared to NC. Supplementary Figure 1. Boxplots for the 13 proteins that differed between SMC and NC. Supplementary Figure 2. Boxplots for the 10 proteins that differed between SMC and PMC. [file 13024_2023_677_MOESM1_ESM.docx]
